# MultiScaleSleepNet: A Hybrid CNN–BiLSTM–Transformer Architecture with Multi-Scale Feature Representation for Single-Channel EEG Sleep Stage Classification

**DOI:** 10.3390/s25206328

**Published:** 2025-10-13

**Authors:** Cenyu Liu, Qinglin Guan, Wei Zhang, Liyang Sun, Mengyi Wang, Xue Dong, Shuogui Xu

**Affiliations:** 1 China-UK Low Carbon College, Shanghai Jiaotong University, Shanghai 200240, China; cenyu_liu@sjtu.edu.cn (C.L.); tsinglinguan@sjtu.edu.cn (Q.G.); zhangv12@sjtu.edu.cn (W.Z.); liyang_sun@sjtu.edu.cn (L.S.); wangmengyi@sjtu.edu.cn (M.W.); 2 Shanghai Changhai Hospital, Shanghai 200433, China

**Keywords:** automatic sleep stage classification, EEG, multi-head attention, temporal-spectral fusion, hybrid architecture

## Abstract

Accurate automatic sleep stage classification from single-channel EEG remains challenging due to the need for effective extraction of multiscale neurophysiological features and modeling of long-range temporal dependencies. This study aims to address these limitations by developing an efficient and compact deep learning architecture tailored for wearable and edge device applications. We propose MultiScaleSleepNet, a hybrid convolutional neural network–bidirectional long short-term memory–transformer architecture that extracts multiscale temporal and spectral features through parallel convolutional branches, followed by sequential modeling using a BiLSTM memory network and transformer-based attention mechanisms. The model obtained an accuracy, macro-averaged F1 score, and kappa coefficient of 88.6%, 0.833, and 0.84 on the Sleep-EDF dataset; 85.6%, 0.811, and 0.80 on the Sleep-EDF Expanded dataset; and 84.6%, 0.745, and 0.79 on the SHHS dataset. Ablation studies indicate that attention mechanisms and spectral fusion consistently improve performance, with the most notable gains observed for stages N1, N3, and rapid eye movement. MultiScaleSleepNet demonstrates competitive performance across multiple benchmark datasets while maintaining a compact size of 1.9 million parameters, suggesting robustness to variations in dataset size and class distribution. The study supports the feasibility of real-time, accurate sleep staging from single-channel EEG using parameter-efficient deep models suitable for portable systems.

## 1. Introduction

### 1.1. Introduction to Sleep Stage Classification

Sleep occupies approximately one-third of the human lifespan and is indispensable to both physical and mental health [1]. Adequate sleep improves cognitive performance and overall well-being [2,3], whereas critical biological processes such as memory consolidation, cellular repair, and cerebral development occur predominantly during sleep [4,5,6,7]. Conversely, sleep deprivation is associated with impaired cognition, immune suppression, and dysregulation of memory, emotion, and metabolism [8,9,10,11,12]. Although the health consequences of sleep issues are well-documented, their wider socioeconomic implications are only now receiving sustained attention. A multinational study across 16 countries in Europe, North America, and Australia found that chronic insomnia affects over 8% of adults, resulting in annual GDP losses of 0.64–1.31% [13]. Further evidence confirms that insufficient or fragmented sleep not only compromises daily emotional stability but also exacerbates productivity declines and societal economic burdens [14]. Given these health and economic repercussions, accurate sleep stage classification is essential for diagnosing sleep disorders and optimizing their management.

**Figure 1:**
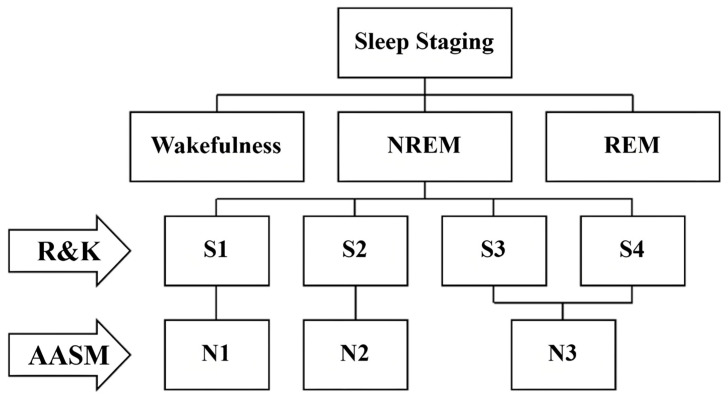
R&K rules and AASM standard guideline on sleep staging.

Polysomnography (PSG) serves as the clinical gold standard for sleep stage classification. It records electroencephalography (EEG), electrooculography (EOG), and electromyography (EMG), providing the basis for separating sleep into non-rapid eye movement (NREM) and rapid eye movement (REM) periods [15]. Following standardized guidelines, including the Rechtschaffen and Kales (R&K) rules [16] and the American Academy of Sleep Medicine (AASM) [17] guideline, specialists manually annotate each 30 s PSG epoch into specific stages. The R&K rules define six sleep stages: wakefulness (Wake), four NREM stages (S1–S4), and REM. Stage S1 represents light sleep, S2 marks a transitional phase, and S3–S4 correspond to deep sleep, also termed slow-wave sleep (SWS). The REM stage, characterized by rapid ocular movements, muscle atonia, and low-voltage mixed-frequency EEG that resembles wakefulness, accounts for 20–25% of adult sleep. In 2007, the AASM merged stages S3 and S4 into a single deep sleep stage (N3), as illustrated in Figure 1.

**Figure 2:**
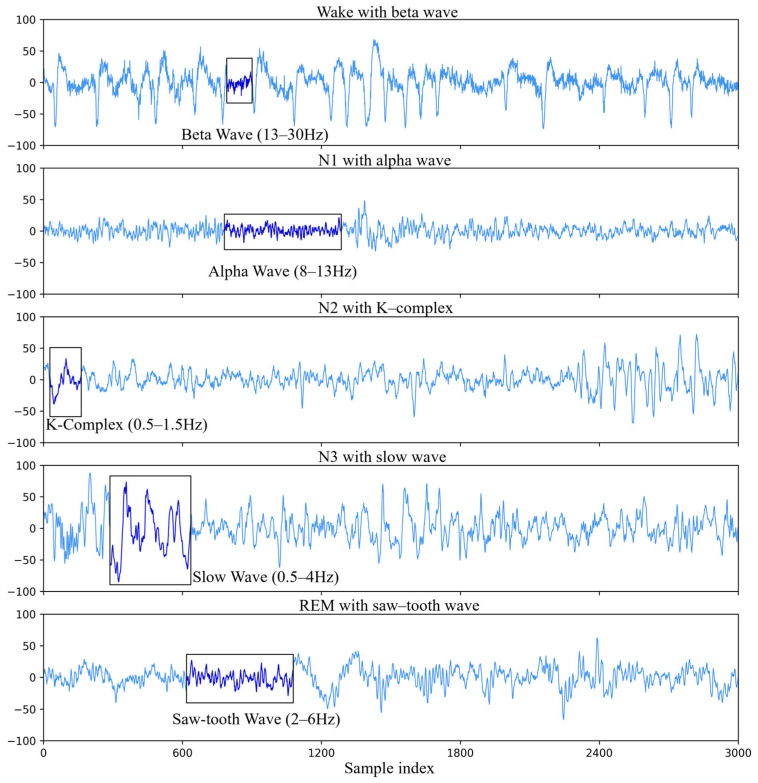
Examples of characteristic EEG signal features across sleep stages from time domain.

Distinct EEG waveforms characterize each sleep stage, as summarized in Table 1. Wakefulness is dominated by β waves, whereas α waves appear during eye closure or meditative states. The transition from the wakefulness stage to light sleep (N1) involves attenuation of α activity and emergence of low-amplitude θ waves. The N1 stage is brief and transitions to N2, which is identified by sleep spindles and K-complexes. Subsequent reductions in cortical activity lead to deep sleep (N3), characterized by high-amplitude δ waves. Finally, REM sleep is distinguished by sawtooth waves in the EEG and rapid ocular oscillations detectable via EOG. Representative time-domain traces for each stage are shown in Figure 2.

### 1.2. Related Work

Traditional sleep stage classification depends on expert visual interpretation of PSG data, which is labor-intensive, time-consuming, costly, and vulnerable to inter- and intra-rater variability that often produces inconsistent results [18]. This manual approach has been proven inefficient for large-scale or real-time applications [19].

To address these challenges, researchers have developed automated sleep stage classification methods that leverage advanced EEG signal processing and machine learning techniques. These methods reduce dependence on manual feature engineering and mitigate the logistical constraints associated with PSG setups [20]. Recent studies have shown particular interest in single-channel EEG augmented with adaptive noise-reduction techniques, a strategy that supports scalable deployment while preserving clinical accuracy [21].

Automated approaches fall into two broad categories. Traditional approaches depend on manually engineered features paired with supervised classifiers such as support vector machine (SVM) and random forest classifiers, whereas deep learning employs neural networks to autonomously extract PSG-derived features through end-to-end learning frameworks.

Early studies adhering to the traditional paradigm emphasized manual feature engineering combined with classical algorithms. For example, Al-Salman et al. [22] applied wavelet transforms to extract time-frequency features from EEG signals, while Abdulla et al. [23] modeled EEG epochs as correlation networks integrated with community detection algorithms. Satapathy et al. [24] enhanced feature optimization by coupling the ReliefF algorithm with an AdaBoost-augmented random forest classifier. Subsequent efforts explored feature refinement strategies, including sliding window statistical mapping [25], principal component analysis (PCA)-based dimensionality reduction [26], and frequency-domain feature extraction using band-pass filters [27]. Despite their interpretability, these methods suffered from limited generalizability due to their reliance on domain-specific expertise, ultimately hindering scalability in large-scale applications.

Deep learning has advanced sleep stage classification by automating feature extraction. Early innovations, such as SleepEEGNet [28], leveraged convolutional neural networks (CNNs) to capture time-invariant spatial patterns in EEG data, while Humayun et al. [29] demonstrated the effectiveness of residual CNNs for raw EEG signal processing. To address temporal dependencies, SeqSleepNet [30] proposed hierarchical architectures combining RNNs, while CSCNN-HMM [31] incorporated hidden Markov models (HMMs) to model state transitions. Heng et al. [32] further advanced temporal modeling with bidirectional gated recurrent units (GRUs) enhanced by attention mechanisms. Subsequent studies refined feature aggregation: AttnSleep [33] fused multi-resolution CNNs with transformer encoders, and XSleepNet [34] dynamically integrated time-frequency representations. Recent developments include transformer-in-transformer architectures [35] for joint local–global dependency learning and multi-task frameworks incorporating channel attention modules [36]. While CNNs and RNNs excel at spatial and temporal feature extraction, hybrid models often face trade-offs between computational efficiency and temporal granularity. Attention mechanisms enhance feature prioritization but necessitate large datasets to prevent overfitting.

Recent research has increasingly focused on multichannel data fusion and transformer-based architectures. MultiChannelSleepNet [37] employed transformer encoders with layer normalization to integrate cross-channel features. Kumar et al. [38] addressed class imbalance through supervised contrastive learning paired with self-attention mechanisms, while Zhao et al. [36] improved cross-dataset generalization using multi-task learning. Discrete Cosine Transform (DCT)-augmented EEGNet [39] optimized multichannel analysis by enhancing spectral domain features. Despite their advantages, multichannel methods escalate computational costs and necessitate strategies to mitigate channel redundancy, highlighting the need for efficient feature selection mechanisms.

Earlier approaches, in contrast to modern hybrid systems, often employed architectures specialized for either spatial or temporal feature extraction. Models such as TimeNet and Temporal Convolutional Networks (TCNs) prioritized convolutional operations for local pattern recognition but lacked mechanisms to capture long-range dependencies. Conversely, BiLSTM-based frameworks, exemplified by SleepEEGNet [28], excelled at sequence modeling but had limited ability to integrate frequency-domain features due to their sequential nature. Even hybrid systems like CSCNN-HMM [31], which integrated HMMs to model sleep stage transitions, omitted dynamic feature weighting via attention mechanisms. Similarly, AttnSleep [33] introduced attention-based aggregation but relied on a dual-branch architecture, potentially limiting its ability to holistically model multi-resolution time-frequency interactions.

To overcome these limitations, this study introduces novel advancements in feature extraction, temporal dependency modeling, and transformer-based architecture design to improve representation learning for sleep stage classification. Departing from existing approaches that treat time- and frequency-domain features in isolation or employ restrictive architectures, our model employs parallel convolutional branches to holistically capture diverse temporal and spectral patterns. Specifically, a Fast Fourier Transform (FFT)-based spectral analysis branch and multi-scale convolutional branches are designed to extract complementary time-frequency features from EEG epochs, enhancing detection of fine-grained signal characteristics. For temporal modeling, the framework integrates a lightweight transformer module with a bidirectional long short-term memory (BiLSTM) network, forming a hybrid encoder. This architecture synergistically combines convolutional operations for local feature extraction, sequential modeling for long-range dependencies, and attention mechanisms for dynamic feature weighting. The BiLSTM component further stabilizes gradient propagation during training while bolstering classification robustness.

The main contributions of this work are as follows:

1. Multi-scale temporal–spectral fusion: a hybrid architecture integrating FFT-based spectral analysis with multi-resolution convolutional branches was developed, achieving comprehensive temporal–spectral feature integration through physiologically-aligned kernel configurations.
2. Hybrid long-range dependency modeling: bidirectional LSTM networks were synergistically combined with transformer self-attention mechanisms, establishing hierarchical temporal context modeling from local sleep transitions to global stage progression patterns.
3. Unified dynamic feature weighting: cross-domain attention mechanisms were implemented to dynamically recalibrate feature significance across concatenated temporal–spectral representations, enabling adaptive fusion of multi-modal sleep characteristics.

The remainder of this paper is organized as follows: Section 2 details the methodology, including the proposed architecture and its operational principles. Section 3 describes experimental protocols, datasets, and results. Section 4 provides a comparative analysis against state-of-the-art methods and discusses performance outcomes. Finally, Section 5 synthesizes key conclusions, evaluates clinical and technical implications, and outlines future research directions.

## 2. Materials and Methods

### 2.1. Methodology

#### 2.1.1. Model Framework

**Figure 3:**
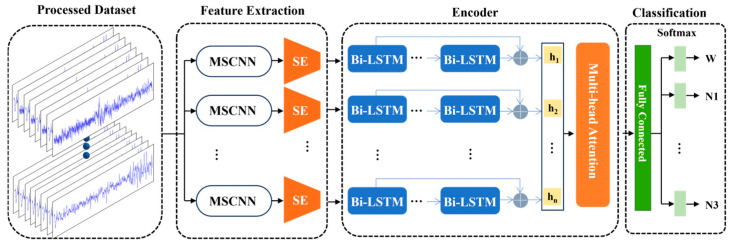
Framework of proposed model.

Figure 3 illustrates the proposed architecture consisting of three sequential modules: (i) multi-scale feature extraction, (ii) sequence context encoding, and (iii) classification. The network accepts a 30 s EEG epoch sampled at 100 Hz (input dimension of 3000 × 1) and outputs one-hot encoded labels for five sleep stages: Wake (W), N1, N2, N3, and REM.

The feature extractor employs a multi-scale convolutional neural network (MSCNN) to capture both time-domain patterns and frequency-domain characteristics. To address the limitations of CNNs in modeling temporal relationships, the sequence context encoder (SCE) integrates a bidirectional LSTM (BiLSTM) layer with a multi-head attention (MHA) mechanism. This combination facilitates the learning of hierarchical, long-range dependencies. The final classifier consists of a fully connected layer followed by temperature-scaled softmax activation. Training uses focal loss with label smoothing to counter class imbalance. Implementation details for each block follow.

#### 2.1.2. Feature Extraction

##### Multiscale Convolutional Neural Network

**Figure 4:**
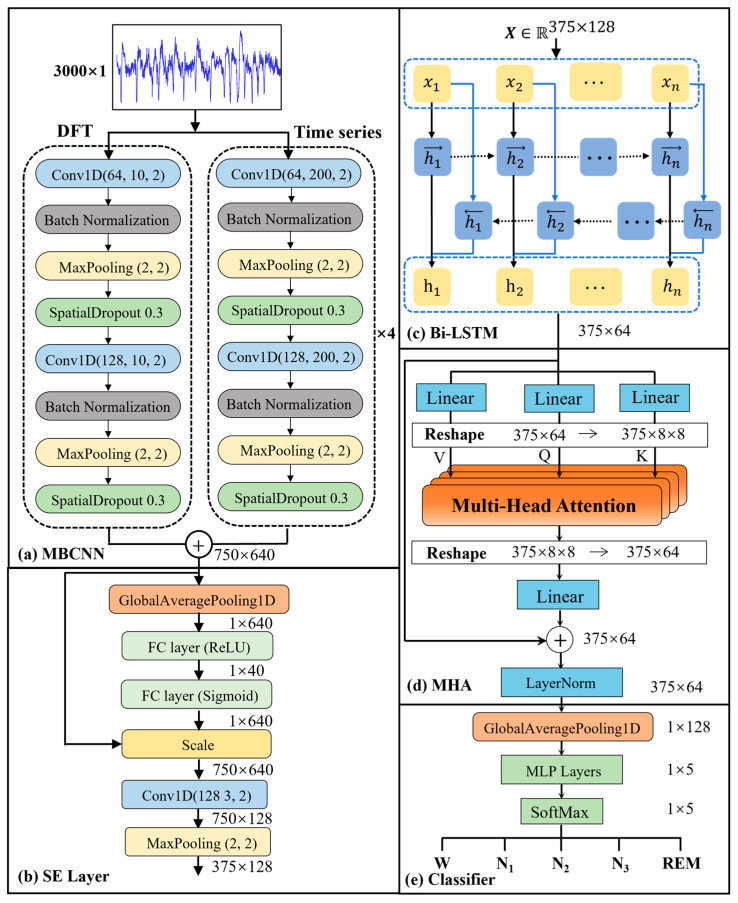
Detailed architecture of MultiScaleSleepNet: (**a**) multiscale convolutional neural network for EEG feature extraction; (**b**) feature map reweighting using the squeeze-and-excitation mechanism; (**c**) bidirectional long short-term memory (BiLSTM) processing block; (**d**) multi-head attention mechanism; (**e**) sleep stage classification with temperature-scaled softmax activation.

The proposed MSCNN architecture integrates both time-domain and frequency-domain representations to comprehensively model sleep EEG dynamics. As shown in Figure 4a, the network consists of five parallel branches: four time-domain convolutional branches targeting specific neurophysiological frequency bands, and one frequency-domain branch for spectral analysis using the discrete Fourier transform (DFT). Raw EEG signals x(t) are transformed into magnitude spectra using DFT:

(1) $$X\left[k\right]=\sum_{n=0}^{N-1}x\left[n\right]exp\left(-j\frac{2\pi kn}{N}\right),k=0,1,\ldots ,N-1$$

where $X\left[k\right]$ represents the frequency component at index k and *N* corresponds to a 30 s epoch sampled at 100 Hz (with dimensions $R^{3000\times 1}$). These magnitude spectra are then processed by 1D convolutional layers to extract stage-related frequency features.

The time-domain branches employ kernel sizes of 200, 25, 13, and 8 samples to align with the characteristic oscillatory periods of the *δ* (0.5–4 Hz), *θ* (4–8 Hz), *α* (8–13 Hz), and *β* (13–30 Hz) bands, respectively. Given a sampling frequency $f_{s}$ (Hz), a kernel of length K spans a temporal window $T=K/f_{s}$. Using f=1/T, the lowest frequency component that can be stably characterized by that kernel is approximated by $f_{min}\approx f_{s}/K$. For $f_{s}=100\;Hz,$ the four kernels yield $f_{min}\approx \left\{0.50,\;4.00,\;7.69,\;12.5\right\}\;Hz$, targeting frequency components corresponding to *δ*, *θ*, *α*, and *β* bands. Each branch thus acts as a learnable band-pass filter tuned to physiologically meaningful oscillations. Each branch further comprises two convolutional blocks (1D convolution, batch normalization, ReLU, spatial dropout, and max pooling), which enhance local temporal discrimination while suppressing noise and redundancy. Formally, the convolution operation in branch i can be expressed as

(2) $$y_{i}\left(t\right)=\sigma \left(\left(x\times w_{i}\right)\left(t\right)+b_{i}\right),\;i\in \left\{\delta ,\;\theta ,\;\alpha ,\;\beta ,\;DFT\right\}$$

where *x* is the input EEG sequence, $w_{i}$ is the branch-specific kernel, $b_{i}$ is the bias term, and σ(·) denotes the activation function. The fused multiscale representation is obtained by channel-wise concatenation,

(3) $$Y_{MSCNN}={⊕}_{i=1}^{5}y_{i}$$

which preserves complementary sub-band information for downstream sequence modeling. This architecture optimizes localized temporal feature extraction while suppressing high-frequency noise. In practice, Fast Fourier Transform (FFT) acceleration lowers complexity from $O(N^{2})$ to O(NlogN) [40]. Max pooling reduces spatial dimensions for efficiency [41], while spatial dropout mitigates overfitting [42]. Finally, multi-scale fusion concatenates all branches along the channel axis to produce the feature map ${Y_{MSCNN}\in R}^{750\times 640}$.

##### Squeeze-and-Excitation (SE) Mechanism

To address channel redundancy in the fused multi-scale representation, a squeeze-and-excitation (SE) block is applied after concatenating the branches. Given an input feature map $U\in R^{T\times C}$, where T = 750 is the number of time steps, C = 640 is the number of feature channels, and U is $Y_{MSCNN}$, the SE module comprises three stages: squeeze, excitation, and recalibration. In the squeeze step, temporal information is aggregated into a channel descriptor Z through global average pooling along the temporal axis:

(4) $$z_{c}=\frac{1}{T}\sum_{t=1}^{T}u_{c}\left(t\right),\;Z\in R^{C}\;$$

where $z_{c}$ is the descriptor of channel *c*. During excitation, the network learns adaptive channel weights via a two-layer fully connected gating mechanism with a reduction ratio *r* = 16.

(5) $$S=\sigma \left(W_{2}\cdot \delta \left(W_{1}\cdot Z\right)\right),\;\left\{\begin{array}{c} W_{1}\in R^{C/r\times C}\\ W_{2}\in R^{C\times C/r} \end{array}\right.\;$$

where δ(·) and σ(·) represent ReLU and sigmoid activations, respectively. In the recalibration stage, the weights *S* are applied elementwise to the original feature map to produce the rescaled output $\tilde{U}$:

(6) $${\tilde{u}}_{c}\left(t\right)=s_{c}\cdot u_{c}\left(t\right),\;\tilde{U}\in R^{375\times 128}\;$$

#### 2.1.3. Sequence Context Encoder

The encoder combines BiLSTM units with an MHA block. BiLSTM propagates hidden states in both forward and backward directions, yielding context-rich representations of the input sequence. MHA then assigns adaptive importance weights by means of scaled dot product operations. Residual links and subsequent layer normalization help to maintain stable gradients. Because this design integrates multi-scale features and attention-guided temporal context within a single module, no separate decoder is required and end-to-end sequence learning is achieved.

##### Bidirectional LSTM Unit

To model long-range sleep stage transitions, we implement a BiLSTM network, which contains four functional components: input layer, forward and backward hidden layers, cell state, and output layer, as shown in Figure 4c. This bidirectional design processes input sequences through complementary temporal passes, allowing simultaneous learning of forward and reverse contextual relationships. The hidden states are computed as follows:

(7) $$\vec{h_{t}}=BiLSTM\left(x_{t},{\vec{h}}_{t-1}\right)$$

(8) $$\overset{\leftarrow }{h_{t}}=BiLSTM\left(x_{t},{\overset{\leftarrow }{h}}_{t-1}\right)$$

(9) $$h_{t}=Concat\left(W_{\vec{h_{t}}}\vec{h_{t}},\;W_{\overset{\leftarrow }{h_{t}}}\overset{\leftarrow }{h_{t}}\right)+b_{t}$$

where $x_{t}\in R^{375\times 64}$ is the input vector at timestep *t*, and $\vec{h_{t}}$ and $\overset{\leftarrow }{h_{t}}$ denote forward and backward hidden states, respectively. The sequence length equals 375 samples and the hidden dimension equals 64. Compared with bidirectional gated recurrent units, BiLSTM can model longer dependencies due to its three-gate structure (input, forget, and output gates) exercising finer control over information flow, which is suitable for capturing polysomnographic patterns [43].

##### Attention Mechanism

To refine feature representations after multi-scale convolutional extraction and BiLSTM-based temporal modeling, we implement a multi-head attention module. The BiLSTM output matrix $X\in R^{375\times 64}$ is mapped into query (Q), key (K), and value (V) matrices through learnable linear transformations. For each of the $h_{a}$ parallel attention heads (8 heads in the proposed model), scaled dot product attention calculates alignment scores:

(10) $$Attention\left(Q,K,V\right)=softmax\left(\frac{QK^{T}}{\sqrt{d_{k}}}\right)V$$

The outputs of all attention heads are concatenated and linearly projected to synthesize multi-head representations:

(11) $$MHA\left(X\right)=Concat\left({head}_{1},\ldots ,{head}_{h_{a}}\right)W^{O},\;W^{Q}\in R^{h_{a}d_{v}\times d}$$

This design enables attention across multiple subspaces, allowing dynamic prioritization of salient temporal features and modeling complex dependencies between time steps [44]. To stabilize training, residual connections combine the original BiLSTM output X with the MHA output $MHA\left(X\right)$, followed by layer normalization:

(12) $$X_{norm}=LayerNorm\left(X+MHA\left(X\right)\right)$$

Finally, global average pooling aggregates temporal features from $X_{norm}$ into sequence-level representations, which are processed by a dropout-regularized fully connected layer for classification.

#### 2.1.4. Classification

Following sequence encoding, the normalized feature matrix $X_{norm}\;\in R^{375\times 64}$ is produced by multi-head attention with residual fusion and then forwarded to the classification module. A GlobalAveragePooling1D layer collapses the temporal dimension, producing a fixed-length vector that condenses the entire sequence context. This vector enters a multilayer perceptron (MLP) with one or more fully connected layers, each followed by dropout to mitigate over-fitting. Temperature scaling is applied to the resulting logits to improve probability reliability without altering class rankings, and a subsequent softmax activation converts the calibrated logits into class probabilities. The network finally outputs a one-hot vector that indicates one of the five sleep stages.

#### 2.1.5. Optimization Strategies

To improve robustness on an imbalanced dataset and to stabilize training, two complementary techniques are incorporated: (1) focal loss with label smoothing, and (2) a cosine annealed learning rate schedule. Together, these measures enhance convergence speed, confidence calibration, and overall classification accuracy.

##### Learning Rate Schedule

Training uses Adam [45] with cosine annealing strategy [46]. Adam’s adaptive moment estimates reduce hyper-parameter sensitivity, while cosine annealing periodically resets the step size, helping the optimizer escape suboptimal minima. The learning rate at epoch *e* is

(13) $$LR\left(e\right)=\frac{\eta_{0}}{2}\left(1+\cos\left(\frac{\pi \cdot e}{N_{epochs}}\right)\right)$$

where $\eta_{0}$ is the initial learning rate and $N_{epochs}$ denotes total training epochs.

##### Loss Function

To counteract class imbalance, we adopt a focal loss function with label smoothing, as introduced by Lin et al. [47]:

(14) $$Loss\left(y_{true},y_{pred}\right)=-\sum_{i=1}^{N}\left(y_{true,i}^{smoothed}\cdot \log\left(y_{pred,i}\right)\cdot \alpha {\left(1-y_{pred,i}\right)}^{\gamma }\right)$$

where $y_{true}^{smoothed}$ represents the smoothed ground-truth labels, and α and γ are hyperparameters controlling the contribution of hard-to-classify examples. Setting γ > 0 reduces the loss weight for well-classified samples, directing focus toward misclassified instances. By dynamically downweighting easy samples, this formulation mitigates bias toward majority classes while improving generalization.

##### Softmax Activation

Output probabilities are calibrated using a temperature parameter τ in the softmax activation:

(15) $$y_{pred,i}=\frac{\exp\left(z_{i}/\tau \right)}{\sum_{j=1}^{N}\exp\left(z_{j}/\tau \right)}\;$$

where $z_{i}$ denotes the logit for class *i*. Temperature scaling preserves class rankings while smoothing overconfident predictions, enhancing reliability in imbalanced scenarios.

### 2.2. Experiments

#### 2.2.1. Datasets

The proposed model was evaluated on three benchmark datasets: the original PhysioNet Sleep-EDF [48] and its expanded version, Sleep-EDF Expanded (Sleep-EDFx) [49], and Sleep Heart Health Study (SHHS) [50,51]. These datasets comprise 61 and 197 full-night PSG recordings, respectively, organized into two cohorts:

- Sleep Cassette (SC): 153 recordings from medication-free Caucasian adults (age range 25 to 101 years) collected during a 1987–1991 observational study investigating age-related sleep physiology;
- Sleep Telemetry (ST): 44 recordings from 22 healthy Caucasian participants (gender-balanced) acquired during a 1994 pharmacological study analyzing the impact of temazepam on sleep macroarchitecture.

Each recording includes two complementary data modalities:

- PSG Files: These files contain full-night neurophysiological signals, including bipolar EEG channels (Fpz–Cz and Pz–Oz), horizontal electrooculography (EOG), submental electromyography (EMG), and event markers;
- Hypnogram Files: These files include expert-curated sleep stage annotations aligned with the PSG data, including Wake (W), REM (REM), NREM stages 1–4 (S1–S4), Movement Time (M), and unscored segments.

The SHHS dataset includes 6441 subjects with various conditions, including pulmonary, cardiovascular, and coronary diseases.

All hypnograms were manually scored by certified sleep experts following R&K rules [16]. In line with AASM guidelines [17] and prior research [28], we merged S3 and S4 into a single SWS class and excluded M and unscored segments from analysis.

#### 2.2.2. Data Preprocessing

**Figure 5:**
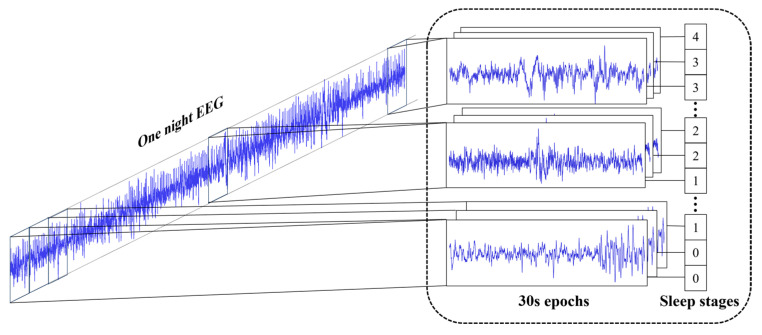
Data segmentation and labeling.

Single-channel EEG from the frontopolar–central (Fpz–Cz, Sleep-EDF/EDFx) or central–mastoid (C4–A1, SHHS) derivation was segmented into 30 s epochs; at sampling rates of 100 Hz and 125 Hz, each epoch contained 3000 and 3750 samples, respectively. As shown in Figure 5, single-channel EEG signals were segmented into 30 s epochs (3000 samples at 100 Hz or 3750 samples at 125 Hz). The Fpz–Cz channel was selected primarily for two reasons. First, this choice ensures consistency with widely adopted baseline studies in sleep staging (e.g., [28,33]), which supports fair and reproducible comparisons. Second, as a bipolar derivation, Fpz–Cz captures broad cortical activity with high signal quality and minimal muscle artifact interference [15,49], making it a representative and practical choice for single-channel EEG analysis. Epoch labels were mapped to five classes: W: 0, N1: 1, N2: 2, N3: 3, and REM: 4. Besides data segmentation, no additional preprocessing (e.g., filtering, artifact removal, or normalization) was applied, as the raw segmented signals were used directly to preserve the original physiological characteristics and align with the data format of the public dataset referenced. The class distribution for both datasets is summarized in Table 2.

#### 2.2.3. Experimental Setup

The framework was implemented in Python 3.8.0 using TensorFlow. To address inherent class imbalance (particularly underrepresented N1, REM, and N3 stages), we integrated focal loss with label smoothing and class weighting during training. All splits were performed at the subject level to prevent any overlap of subjects across training, validation, and test sets. For Sleep-EDFx, both nights from the same subject were kept together in the same split. We first grouped all 30 s epochs by subject (and by recording/night when applicable), then sampled 80%/10%/10% of subjects for training/validation/test sets using a fixed random seed (42). Training used the Adam optimizer with an initial learning rate of 0.001 and batch size of 128. Each epoch input had dimensions of 3000 × 1 per epoch, resulting in batch input dimensions of 128 × 3000 × 1.

To ensure a fair and meaningful comparison with existing state-of-the-art models, we adhered to the following consistent experimental protocols throughout our study:

- Datasets and Channels: All experiments were conducted on the standard versions of the Sleep-EDF, Sleep-EDFx, and SHHS datasets, employing the single-channel Fpz–Cz or C4–A1 derivation. This choice aligns with the most commonly adopted settings in the literature against which we compare.
- Data Preprocessing: We applied identical preprocessing steps: segmentation into 30 s epochs, no additional filtering or artifact removal, and mapping of sleep stages to five classes (W, N1, N2, N3, REM) in accordance with AASM guidelines.
- Data Splitting: A subject-wise split strategy was strictly followed to prevent data leakage, ensuring that all epochs from the same subject were contained within either the training, validation, or test set.

While minor, uncontrolled variations in evaluation protocols (e.g., specific random seeds for splitting) may exist across different studies, the performance improvements reported in this work are primarily attributed to the proposed architectural advances under this aligned experimental framework.

#### 2.2.4. Evaluation Metrics

To comprehensively evaluate the performance of the proposed method, we used multiple evaluation metrics, including overall accuracy (ACC), precision (PR), recall (RE), Cohen’s kappa coefficient (κ), and the F1 score, defined as follows:

(16) $$ACC=\left(\frac{TP+TN}{TP+FP+FN+TN}\right)\times 100\%$$

(17) $$PR=\left(\frac{TP}{TP+FP}\right)$$

(18) $$RE=\left(\frac{TP}{TP+FN}\right)$$

(19) $$F1=\left(\frac{2\times PR\times RE}{PR+RE}\right)$$

(20) $$\kappa =\frac{P_{0}{-P}_{e}}{1-P_{e}}$$

True Positive (TP) refers to the sleep stage instances correctly classified by the model. True Negative (TN) refers to instances correctly identified as not belonging to a specific sleep stage. False Positive (FP) refers to instances incorrectly predicted as belonging to a sleep stage, while False Negative (FN) refers to instances misclassified as other sleep stages.

ACC provides global classification performance, while PR and RE quantify prediction reliability and stage-specific detection capability, respectively. The F1-score balances these metrics, particularly critical for imbalanced data. κ evaluates agreement between model predictions and expert annotations, with κ > 0.6 indicating strong concordance based on the Landis–Koch criteria [52]. These metrics objectively assess alignment with ground-truth hypnograms by treating the model as an automated rater.

## 3. Results

### 3.1. Classification Performance of the Proposed Model

The performance of the proposed model was evaluated using class-wise evaluation metrics and confusion matrices, as presented in Table 3, Table 4 and Table 5. The model achieved an overall accuracy of 85.6% on the Sleep-EDFx dataset (Fpz–Cz EEG channel). On the Sleep-EDF dataset, it reached 88.6% accuracy and 84.6% on the SHHS dataset.

**Figure 6:**
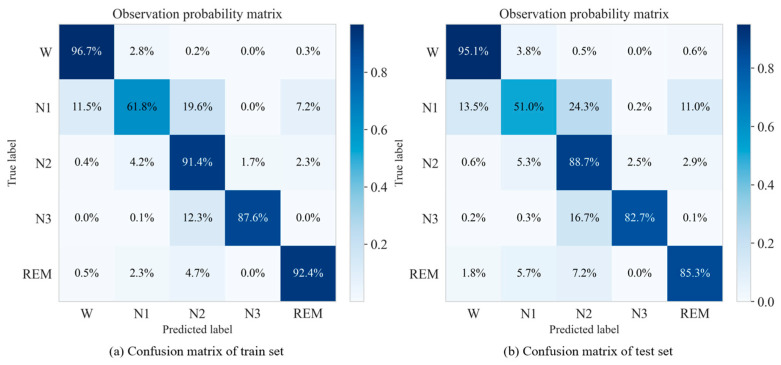
Confusion matrix of training and test sets on the Fpz–Cz channel of the Sleep-EDFx dataset.

To provide a more comprehensive view, the confusion matrix for the Fpz–Cz EEG channel on the Sleep-EDFx dataset, along with probability matrices for both the training and testing sets are shown in Figure 6. The diagonal elements in this matrix represent True Positive (TP) counts, and the matrices illustrate consistency in stage-wise classification across datasets.

The model achieves high recall rates of over 85% for the Wake, N2, N3, and REM stages, confirming its effectiveness in capturing the major electrophysiological signatures of these sleep states. Notably, Wake and N2 stages maintain high generalization performance, with training recall values of 96.7% and 91.4%, and testing recall values of 95.1% and 88.7%, respectively. The recall for N3 declines slightly from 87.6% in training to 82.7% in testing, but remains sufficient for clinically relevant detection of slow-wave sleep. The REM stage shows a more noticeable decrease, from 92.4% to 85.3%, possibly due to inter-individual variability, similar to other sleep stages or the inherent limitations of single-channel EEG in capturing phasic REM features.

The N1 stage continues to pose the greatest classification difficulty, as evidenced by a recall drop from 61.8% in the training set to 51.0% in the testing set. Misclassifications predominantly occur with adjacent stages, with N2 and Wake accounting for 20% and 12% of errors, respectively, and REM contributing another 7%. These results underscore the inherent ambiguity of N1, whose mixed-frequency EEG features overlap with both light sleep and REM. This overlap complicates the classification and is consistent with findings in the polysomnographic literature [18].

Table 6 presents a comprehensive performance comparison between the proposed model MultiScaleSleepNet and established baselines across experimental datasets. Three key observations can be drawn from this comparison.

First, MultiScaleSleepNet achieves consistently higher accuracy while using only single-channel EEG input. It outperforms conventional sequence-to-sequence architectures such as SleepEEGNet [28], XsleepNet2 [34], and AttnSleep [33] across both large-scale (Sleep-EDFx) and small-scale (Sleep-EDF) datasets. Notably, it improves overall accuracy over SleepEEGNet by 5.6% on Sleep-EDFx and 4.4% on Sleep-EDF, confirming its robustness across varying dataset sizes.

Second, the model demonstrates consistent superiority across all evaluation metrics. On Sleep-EDFx, MultiScaleSleepNet achieves relative improvements of 1.6%, 4.3%, 2.9%, 1.0%, 0.6%, and 2.2% in accuracy over XsleepNet2, AttnSleep, SleepContext-Net, CSCNN-HMM, MultiChannel-SleepNet, and Multi-Task Learning, respectively. On Sleep-EDF, the advantages become more pronounced, with gains of 2.3%, 4.2%, 3.8%, 1.4%, and 3.0% over the same models (excluding CSCNN-HMM, which lacks reported results for this dataset). Moreover, despite using only one EEG channel, MultiScaleSleepNet surpasses the performance of MultiChannel-SleepNet, which relies on three-channel input, and achieves the highest macro-F1 scores and kappa coefficients on both datasets.

Third, the class-wise analysis reveals exceptional capability in addressing data imbalance. MultiScaleSleepNet achieves optimal class-wise F1-scores across all sleep stages, particularly excelling in underrepresented stage N1. These results highlight the dual effectiveness of the proposed architecture in mitigating data imbalance while sustaining high overall classification performance.

### 3.2. Hyperparameter Tuning

Hyperparameter optimization played a crucial role in performance enhancement. Sensitivity analysis was performed on dropout rate, learning rate, and batch size to optimize performance. The best configuration (dropout = 0.3, learning rate = 1 × 10^−3^, batch size = 128) achieved the highest accuracy, macro-F1, and kappa across datasets.

**Figure 7:**
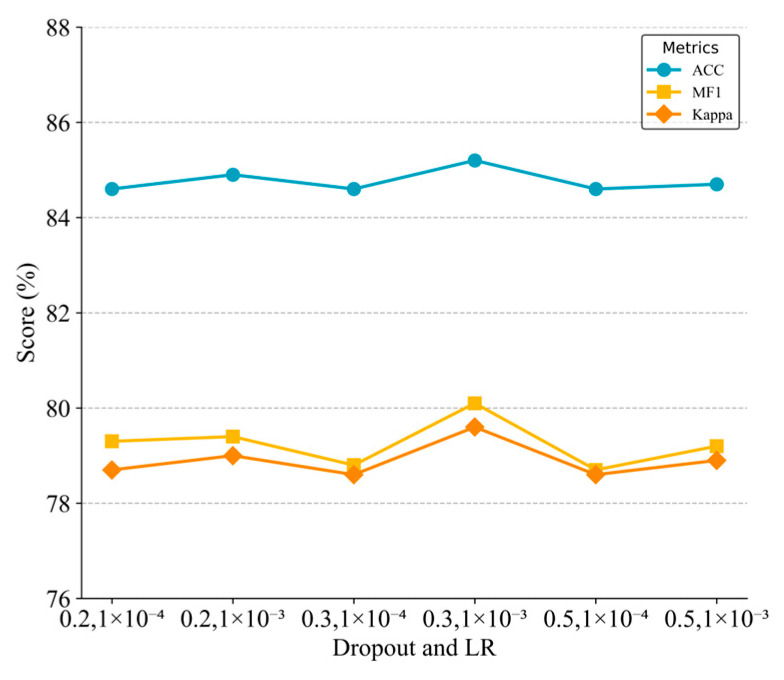
Impact of dropout and learning rate on model performance across evaluation metrics.

We first examined dropout rates (0.2, 0.3, 0.5) and initial learning rates (1 × 10^−3^, 1 × 10^−4^) through repeated trials. As shown in Figure 7, the optimal configuration was a dropout rate of 0.3 and a learning rate of 0.001, achieving peak values for accuracy (85.5%), macro-F1 score (0.82), and kappa coefficients (0.80). A higher dropout of 0.5 led to underfitting and longer training times (approximately 15% increase), due to excessive suppression of temporal features.

**Figure 8:**
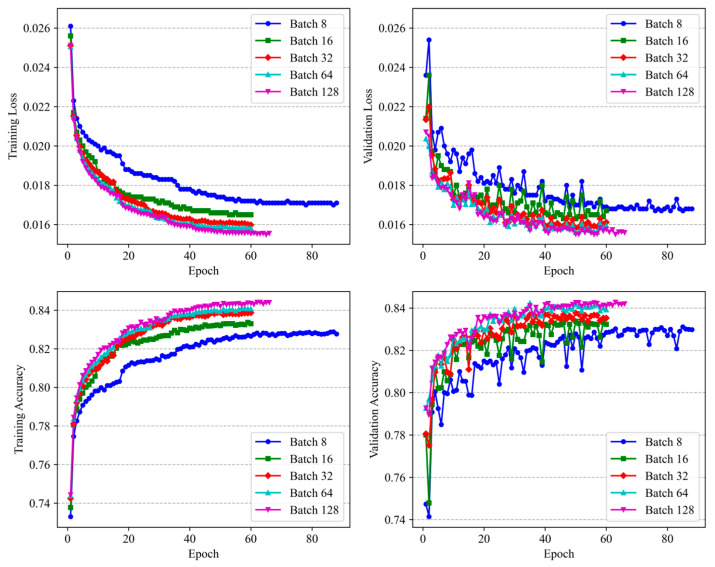
Accuracy and loss curves for different batch sizes on Sleep-EDFx.

Next, the impact of batch size on training dynamics was subsequently examined. As illustrated in Figure 8, larger batch sizes, particularly a size of 128, led to faster convergence, lower training loss, and improved accuracy. The superior performance associated with this configuration is likely due to smoother gradient updates that reduce noise during parameter optimization. Additionally, it enhances the ability to capture long-term temporal dependencies in the time-series data. These advantages contribute to more stable and efficient model training, indicating that a batch size of 128 offers an optimal balance between computational efficiency and generalization capability for this task.

### 3.3. Sleep Stage Prediction Analysis

To assess temporal consistency and classification stability across varying time scales, the model was tested on continuous sequences of 100, 200, and 300 epochs. These sequences were randomly extracted from the continuous recordings of the Sleep-EDFx test set to simulate realistic, arbitrary-length data segments and to evaluate the robustness of our approach without bias towards a specific sequence length. Our model architecture is inherently flexible to variable-length inputs, as it processes each epoch independently through the same feature extraction pipeline without relying on fixed-length temporal context. The final sequence-level accuracy for a given window was then calculated as the proportion of epochs whose predicted labels exactly matched the ground truth across the entire continuous segment.

**Figure 9:**
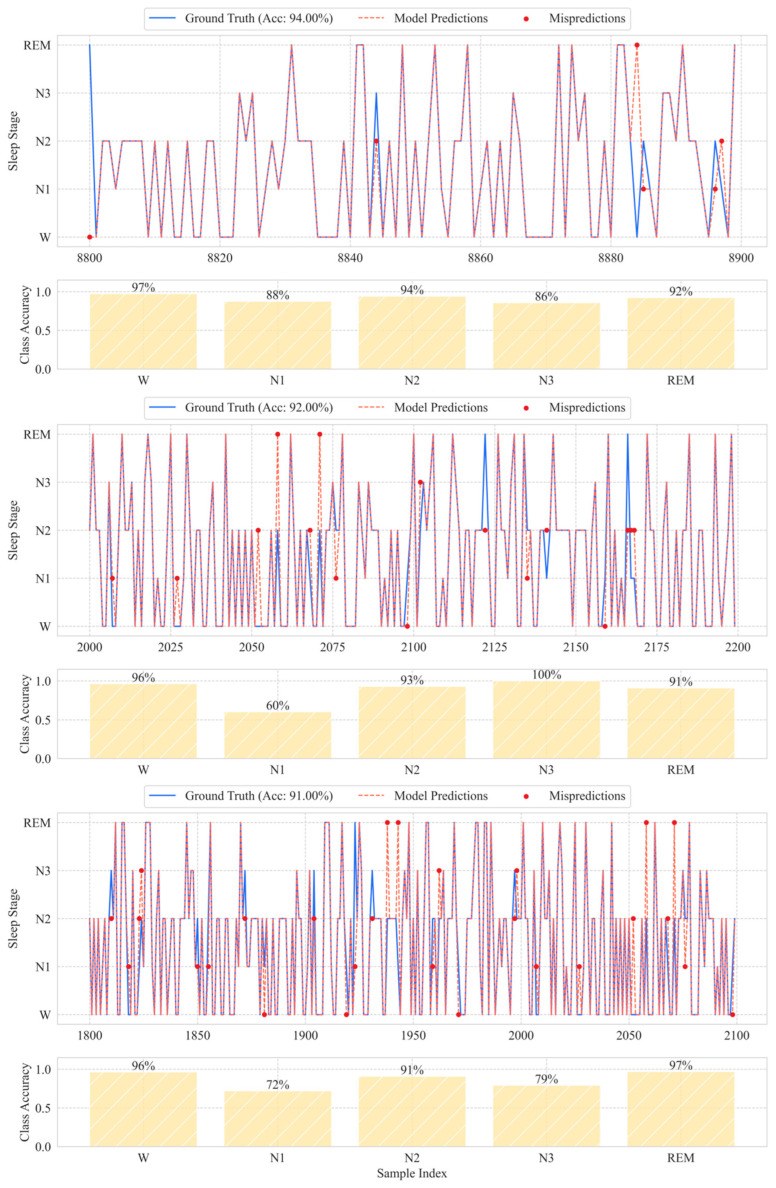
Ground-truth and predicted labels for three random sequences of 100, 200, and 300 contiguous epochs from the Sleep-EDFx test set. Acc indicates sequence-level accuracy. Red dots mark epochs that were misclassified.

As illustrated in Figure 9, the predicted labels closely aligned with expert annotations for all window lengths. Accuracy remained high, slightly decreasing from 94% for 100-epoch windows, to 91% for 300-epoch windows. Notably, the model achieved 88% accuracy in identifying the N1 stage in 100-epoch segments, demonstrating resilience in detecting this challenging stage.

Error analysis revealed two principal trends. First, 65% of misclassifications occurred at stage boundaries (e.g., N1/N2 and N3/REM), reflecting the ambiguity of transitional epochs and inter-rater variability. Second, frequent confusion between N1 and REM was linked to overlapping alpha wave patterns, common to both stages. These observations suggest that model limitations align with recognized clinical challenges and may be mitigated through improved modeling of transition dynamics.

### 3.4. Ablation Experiments

To evaluate the impact of each architectural component, ablation studies were conducted on model variants, as shown in Table 7. The baseline BiLSTM configuration achieved an accuracy of 73.2%, a macro-F1 score of 0.600, and kappa coefficient of 0.619, requiring 258 epochs to converge. This performance highlights the limitations of using temporal modeling alone.

Adding a convolutional backbone improved accuracy by 13.4% to 83.0%, while reducing training time to 210 epochs. The combined CNN-BiLSTM model further improved performance to 84.9% accuracy and a macro-F1 score of 0.795, albeit with a slight increase in convergence time to 261 epochs.

Introducing the squeeze-and-excitation (SE) mechanism improved discriminative capability, resulting in 85.5% accuracy and a macro-F1 of 0.800. However, this enhancement extended training to 356 epochs. The final architecture, which incorporates transformer-based attention with an optimized BiLSTM, achieved peak performance (85.6% accuracy, 0.811 macro-F1, and 0.804 kappa coefficient) and converged in only 200 epochs, reducing training time by 43.8% relative to the SE-enhanced model.

These results confirm three key design principles: (i) multi-scale CNNs enable effective hierarchical spectral decomposition, (ii) BiLSTMs capture bidirectional temporal dependencies, and (iii) attention mechanisms dynamically prioritize important features. The final model offers an effective trade-off between representational power and training efficiency.

## 4. Discussion

MultiScaleSleepNet is a hybrid model comprising 1.9 million parameters that integrates multi-scale convolutional branches, bidirectional LSTM, and lightweight attention for single-channel EEG classification. It achieves high accuracy across the Sleep-EDFx (85.6%), Sleep-EDF (88.6%), and SHHS (84.6%) datasets, with kappa coefficients of 0.80, 0.84, and 0.79, respectively. The model also maintains robust F1-scores across the Wake, N2, N3, and REM stages and significantly improves the classification of the diagnostically challenging N1 stage. The consistent performance on the SHHS dataset, which encompasses a broader and more clinically diverse population, further demonstrates the model’s generalizability and potential utility beyond healthy adult cohorts.

When comparing our results to those of earlier studies, it is important to note that although all leading models use CNN backbones for feature extraction from time-series data, their architectures differ significantly. MultiChannel-SleepNet and recent transformer-only models rely on multi-channel recordings or deep attention stacks, which increase hardware costs or degrade sharply when training data fall below 100 nights. In contrast, MultiScaleSleepNet achieves comparable or superior accuracy using only single-channel EEG, suggesting that architectural balance, rather than model size, governs generalization. The 10% and 12% relative F1-score improvements for N1 classification over SleepEEGNet and AttnSleep highlight that its gains arise from architectural design rather than parameter tuning.

Class-wise analysis demonstrates the strong capability of the model in handling data imbalance. MultiScaleSleepNet achieves competitive F1-scores across all sleep stages, with particularly outstanding performance in identifying the underrepresented yet clinically critical N1 stage, and exhibits high precision in distinguishing REM sleep from wakefulness. The improved capability in N1 sleep detection holds significant clinical relevance. Accurate identification of N1 sleep is crucial for diagnosing insomnia, as patients often present with prolonged N1 duration and frequent stage transitions. By utilizing specially optimized theta-band convolutional kernels, our model effectively captures the low-amplitude, mixed-frequency EEG characteristics of N1 sleep, thereby providing a more reliable basis for clinical assessment. Meanwhile, the performance of the model in discriminating REM sleep from wakefulness contributes to the screening of REM sleep behavior disorder (RBD). By integrating both tonic and phasic features of REM sleep, the model significantly reduces misclassification between REM and wakefulness, enabling more accurate association of abnormal motor behaviors with REM sleep rather than wakefulness. These results fully demonstrate the dual strengths of the proposed architecture: overcoming the technical challenges of data imbalance while delivering performance improvements with tangible clinical value.

It is worth emphasizing that although earlier studies often attribute performance degradation on small datasets to model over-parameterization, the proposed model maintains robust accuracy under data-constrained conditions. Moreover, contrary to the assumption that multi-lead inputs are necessary for reliable N1 detection, the proposed multi-scale convolutional design demonstrates effective recovery of inter-electrode information using only a single channel. Although MultiScaleSleepNet incorporates five convolutional branches, the total number of parameters remains limited to 1.9 million, which corresponds to approximately one-third of the parameter count of XSleepNet2 (5.8 million) [34]. This compact architecture enhances its suitability for large-scale and cost-effective deployment in both clinical and home-based sleep monitoring scenarios. In addition, the integration of the discrete Fourier transform within the model framework offers a generalizable preprocessing approach that can be readily extended to other types of biosignals, including audio, EOG, and EMG.

## 5. Conclusions

In this study, we introduced MultiScaleSleepNet, a hybrid architecture for automatic sleep staging that learns simultaneously from raw EEG signals and their multiscale spectral representations. The network integrates parallel convolutional branches, bidirectional long short-term memory layers, and multi-head attention to capture both local rhythmic patterns and long-range temporal context from a single EEG channel. Designed for robustness across varying dataset sizes, MultiScaleSleepNet effectively balances its architectural components and mitigates overfitting through shared feature normalization and scheduled dropout.

Ablation studies validate the functional contributions of each module: multi-scale convolutional networks enhance spectral discrimination, BiLSTM layers capture bidirectional temporal dependencies, and attention mechanisms enable adaptive feature weighting. Furthermore, the incorporation of focal loss with label smoothing significantly improves classification of underrepresented stages, particularly the challenging N1 stage.

Experimental results on the Sleep-EDF and Sleep-EDFx datasets demonstrate that MultiScaleSleepNet outperforms existing single-channel baseline models. Its efficient inference enables practical deployment on resource-constrained edge devices and wearable sensors. These findings underscore the potential of our framework to facilitate large-scale, cost-effective sleep monitoring and support both clinical and at-home population health applications. Moreover, future work will focus on validating the generalizability of the model on clinical datasets containing patients with sleep disorders to further assess its utility in real-world diagnostic settings.

## Figures and Tables

**Table 1:** EEG frequency of different bands.

Band	Delta (δ)	K-Complex	Saw-Tooth	Theta (θ)	Alpha (α)	Sleep Spindles	Beta (β)
Frequency Range (Hz)	0.5–4	0.5–1.5	2.0–6.0	4–8	8–13	12–14	13–30

**Table 2:** Number of 30 s epochs for each sleep stage in the processed dataset.

Dataset	W	N1	N2	N3	REM	Total
Sleep-EDF	8285	2804	17,799	5703	7717	42,308
Sleep-EDFx	69,546	24,650	86,884	18,703	33,573	233,356
SHHS	46,319	10,304	142,125	60,153	65,953	324,854

**Table 3:** Class-wise evaluation metrics on the Sleep-EDFx test set (Fpz–Cz channel), which accounts for 10% of the total Sleep-EDFx dataset.

Label	PR	RE	F1	Support
W	0.94	0.95	0.94	6954
N1	0.58	0.51	0.54	2465
N2	0.87	0.89	0.88	8689
N3	0.88	0.83	0.85	1870
REM	0.83	0.85	0.84	3358
ACC			0.86	23,336
κ			0.80	23,336

**Table 4:** Class-wise evaluation metrics on the Sleep-EDF test set (Fpz–Cz channel), which accounts for 10% of the total Sleep-EDF dataset.

Label	PR	RE	F1	Support
W	0.94	0.94	0.94	829
N1	0.58	0.49	0.53	280
N2	0.90	0.92	0.91	1780
N3	0.90	0.93	0.91	571
REM	0.87	0.86	0.87	771
ACC			0.89	4231
κ			0.80	4231

**Table 5:** Class-wise evaluation metrics on the SHHS test set (C4–A1 channel), which accounts for 10% of the total Sleep-EDFx dataset.

Label	PR	RE	F1	Support
W	0.84	0.87	0.85	4675
N1	0.48	0.19	0.28	1102
N2	0.89	0.85	0.87	14,283
N3	0.87	0.90	0.89	6101
REM	0.77	0.89	0.82	6325
ACC			0.85	32,486
κ			0.79	32,486

**Table 6:** Performance comparison of the proposed model with state of the art models.

Methods	Dataset	Over Performance			Per-Class Performance (F1)				
		ACC	MF1	κ	W	N1	N2	N3	REM
SleepEEGNet [28]	Sleep-edfx	80.03	73.55	0.73	91.72	44.05	82.49	73.45	76.06
XsleepNet2 [34]	Sleep-edfx	84.00	77.90	0.78	-	-	-	-	-
AttnSleep [33]	Sleep-edfx	81.3	75.1	0.74	92.0	42.0	85.0	82.1	74.2
SleepContext-Net [53]	Sleep-edfx	82.7	77.2	0.76	92.8	49.0	84.8	80.6	76.4
CSCNN-HMM [31]	Sleep-edfx	84.6	78.0	0.79	93.0	41.0	88.0	85.0	83.0
MultiChannel-SleepNet [37]	Sleep-edfx	85.0	79.6	0.79	94.0	53.0	86.9	81.8	82.6
Multi-Task Learning [36]	Sleep-edfx	83.4	78.6	0.77	92.9	52.6	84.9	78.9	83.7
Proposed Model	Sleep-edfx	85.6	81.1	0.80	94.0	54.0	88.0	85.0	84.0
SleepEEGNet [28]	Sleep-edf	84.26	79.66	0.79	89.19	52.19	86.77	85.13	85.02
XsleepNet2 [34]	Sleep-edf	86.3	80.6	0.81	-	-	-	-	-
AttnSleep [33]	Sleep-edf	84.4	78.1	0.79	89.7	42.6	88.8	90.2	79.0
SleepContext-Net [53]	Sleep-edf	84.8	79.8	0.79	89.6	50.5	88.4	88.5	82.0
MultiChannel-SleepNet [37]	Sleep-edf	87.2	82.0	0.81	92.8	49.1	90.0	89.3	84.8
Multi-Task Learning [36]	Sleep-edf	85.6	81.1	0.80	90.4	53.7	88.3	88.3	85.1
Proposed Model	Sleep-edf	88.6	83.3	0.84	94.0	53.0	91.0	91.0	87.0
SleepEEGNet [28]	SHHS	73.9	68.4	0.65	81.3	34.4	73.4	75.9	77.0
AttnSleep [33]	SHHS	84.2	75.3	0.78	86.7	33.2	87.1	87.1	82.1
Proposed Model	SHHS	84.6	74.5	0.79	85.0	28.0	87.2	89.0	82.2

**Table 7:** Comparative performance evaluation of proposed model and ablation variants on sleep-EDFx dataset.

Components	ACC (%)	Macro-F1	Kappa	Epochs to Converge
BiLSTM (baseline)	73.2	0.600	0.619	258
CNN	83.0	0.767	0.767	210
CNN + BiLSTM	84.9	0.795	0.792	261
+SE Module	85.5	0.800	0.800	356
Proposed Model	85.6	0.811	0.804	200

## Data Availability

Code/weights will be made available on request.
